# Retrospective evaluation *versus* population norms for the measurement of baseline health status

**DOI:** 10.1186/1477-7525-10-68

**Published:** 2012-06-14

**Authors:** Ross Wilson, Sarah Derrett, Paul Hansen, John Langley

**Affiliations:** 1 Injury Prevention Research Unit, Department of Preventive and Social Medicine, University of Otago, PO Box 56, Dunedin, 9054, New Zealand; 2 Department of Economics, University of Otago, Dunedin, New Zealand

**Keywords:** Health-related quality of life (HRQoL), Recall bias, EQ-5D, Population norms

## Abstract

**Background:**

Patient recall or the application of population norms are commonly used methods to estimate (unobservable) health status prior to acute-onset illness or injury; however, both measures are potentially subject to bias. This article reports tests of the validity of both approaches, and discusses the implications for reporting changes in health-related quality of life following acute-onset illness or injury.

**Methods:**

Recalled pre-injury health status and health status at 5- and 12-months post-injury were collected from participants in a prospective cohort study of people injured in New Zealand. Reported post-injury health status was compared with recalled pre-injury status and New Zealand norms for two groups: those who reported having fully recovered, and those who had not.

**Results:**

There was a small but statistically significant difference between pre- and post-injury health state valuations for people who had fully recovered, with recalled pre-injury health status being higher than reported post-injury health. Perceived health status for those who had fully recovered was significantly higher than the population norm.

**Conclusions:**

Retrospective evaluation of health status is more appropriate than the application of population norms to estimate health status prior to acute-onset injury or illness, although there may be a small upward bias in such measurements.

## Background

Generic measures of health status are designed to gauge changes in people’s health status over time such as their recovery from illness or injury. Instruments such as the Health Utilities Index, SF-6D and EQ-5D are used for deriving health state preference values for calculating Quality-Adjusted Life Years (QALYs) for use in economic cost-effectiveness analyses [1]. This article uses the EQ-5D. Developed by the EuroQol Group, the EQ-5D represents health in terms of five dimensions: mobility, self-care, ability to participate in usual activities, pain or discomfort, and anxiety or depression; with three possible responses per dimension (no problems, moderate problems, and extreme problems) [2].

The EQ-5D has been included in national population health surveys in the United Kingdom, Canada, China, Finland, Spain, Denmark, the United States and New Zealand [3,4]. The National Institute for Health and Clinical Excellence (NICE) has recommended the EQ-5D be used in trials and observational studies of health outcomes to provide QALY information about the effects of new treatments [5]. Since 2009, NHS secondary health providers in England have been asked to collect EQ-5D data for four surgical patient groups, pre- and post-operatively, as part of the Patient Reported Outcome Measures (PROMS) initiative [6]. Data have been collected from hundreds of thousands of patients so far [7].

To measure change in health status, information about pre- and post-intervention health is required. Similarly, if the focus is determining health burden borne by groups affected by particular conditions, information about health before and after the onset of the condition is required. However, in studies looking at acute-onset conditions (*e.g.* cancer, stroke, injury), participants are usually recruited only after the health event has occurred. In such cases, researchers tend to adopt one of two approaches. Either they apply population norms to estimate pre-onset health, or they ask participants to ‘recall’ their pre-onset health status. Both approaches have limitations.

Applying population norms as the pre-onset health status may, logically, either under- or over-estimate the true health burden. The former would arise when, in fact, the people affected by a condition such as myocardial infarction were actually in poorer health before the myocardial infarction occurred; the latter when, in fact, the people with, say, traumatic brain injury were in better health than the general population before the injury. Similarly, when applying recalled health status the burden may be over-estimated if participants recall unrealistically high health states.

A study by Watson and colleagues [8] investigated the health status of patients reporting they had “recovered” from injury vis-à-vis their recalled pre-injury status and population norms, using the SF-36, SF-6D and AQoL instruments. No statistically significant differences, or at most only marginal differences, were found between recalled pre-injury health status and “recovered” status one year later, and recalled pre-injury statuses were consistently higher than general population norms. However, this study was restricted to hospitalised patients, and had a small sample size (n = 186), of whom only 61 reported full recovery.

Using a similar method, but with a larger cohort and wider range of injuries, the Prospective Outcomes of Injury Study (POIS) underway in New Zealand provides an opportunity to investigate the validity of using retrospective evaluation or population norms as proxies for pre-onset health valuation using the EQ-5D. This article reports the results of this analysis and discusses the implications for reporting changes in health-related quality of life (HRQoL) following illness or injury.

## Methods

### Data

The Accident Compensation Corporation (ACC) provides universal no-fault insurance for people injured in New Zealand. This article uses data from POIS, a prospective cohort study of individuals, aged between 18 and 64 years, recruited from the ACC entitlement claims register between December 2007 and June 2009 [9,10]. Participants included patients with all injury types, except those whose injuries were a result of self-harm or sexual assault, and covered a wide range of injury severities. Participants (n = 2856) completed a first interview 3.2 months (on average) after injury, with follow-up interviews approximately 5 months (average of 4.6) and 12 (12.3) months after injury. The POIS study received ethical approval from the New Zealand Health and Disability Multi-region Ethics Committee (MEC/07/07/093).

### Measures

Two components of the POIS interview were used to identify “fully recovered” participants. First, participants were asked at the start of the 5-month and 12-month interviews whether they had completely recovered or were still affected by their injury. Second, they were assessed on the 12-item World Health Organization Disability Assessment Schedule (WHODAS 2.0), an instrument developed by the World Health Organization (WHO) to measure disability [11]. The WHODAS rates participants’ difficulty completing a set of 12 activities over the previous 30 days, with five responses options from “None” to “Extreme/Cannot Do.” In all three interviews, participants were asked to complete the WHODAS instrument for their current health, and, in the first (3-month) interview they were asked to complete the WHODAS for the 30 days preceding their injury. We defined participants as having recovered from injury at the 5-month and 12-month interviews if they, in effect, passed both of the tests discussed above – *i.e.* reported having “completely recovered” and having attained at least their pre-injury functioning on all 12 WHODAS items.

At the first interview, participants were asked to complete the EQ-5D with respect to their pre-injury health status. They also did the same with respect to their (current) health status at 5- and 12-months after injury. The New Zealand EQ-5D valuation set [12] was used to convert participants’ health profiles on five dimensions to values on a scale from 0 (death) to 1 (perfect health), with negative values for states considered to be worse than dead.

### Analysis

To test the validity of participants’ retrospective evaluations of their health status, we compared their recalled pre-injury health with their reported health at 5- and 12-months after injury. These comparisons were performed for two groups: participants who reported having fully recovered, and others who reported having not. We hypothesised that for fully-recovered participants if their recalled pre-injury health status is unbiased then it would be the same as their post-injury status.

We also compared participants’ health valuations with population norms from the survey of the New Zealand general population undertaken in 1999 from which the above-mentioned EQ-5D valuation set was derived [12]. Respondents described their own EQ-5D health status, and preference values were then applied from the valuation set. Population norms were calculated as the age- and sex-adjusted average of respondents’ valuations. We hypothesised that if population norms are a valid proxy for the pre-injury health of injured patients then population norms would approximate the health status of POIS participants who reported having fully recovered.

## Results

### General characteristics

As represented on the EQ-5D’s five dimensions, 2842 POIS participants recalled their pre-injury health status at the first interview, and 1475 and 2262 respectively also reported their current health status at the 5- and 12- month post-injury interviews. Fewer participants completed the second (5-month) interview, as many had completed the first interview at the time the second interview was scheduled for, due to unanticipated delays in recruitment and interviewing [10]. *Via* the survey of the general population, 1250 respondents described their health status on the EQ-5D, of whom 964 were aged 18–64 and identified their sex, allowing for construction of adjusted population norms.

Table 1 describes the age, sex, recovery status, health status, and injury types of POIS participants and respondents to the general population survey. POIS participants were on average younger and more likely to be male than those in the general population survey.

**Table 1:** Age, sex, recovery status, and EQ-5D HRQoL of POIS participants and general population survey respondents

	**Pre-injury**	**5-months post-injury**	**12-months post-injury**	**NZ general population survey**
**Total**	2842	1470	2262	1250
**Age:**				
**18-24**	14.4%	13.1%	12.1%	7.0%
**25-34**	20.8%	19.5%	19.5%	13.3%
**35-44**	22.5%	23.5%	22.5%	20.4%
**45-54**	24.5%	26.5%	26.3%	20.2%
**55-64**	17.8%	17.4%	19.6%	16.4%
**65+**	–	–	–	21.0%
**Not reported**	–	–	–	1.8%
**Sex:**				
**Male**	61.4%	60.8%	58.9%	42.7%
**Female**	38.6%	39.2%	41.1%	56.0%
**Not reported**	–	–	–	1.3%
**Recovery Status:**		n = 1248	n = 1937	
**Recovered**		23.0%	36.4%	
**Not recovered**		77.0%	63.6%	
**Injury Type:**				
**Intracranial**	3.7%	3.7%	3.8%	
**Head/neck superficial**	3.6%	3.2%	3.6%	
**Spine dislocation, sprain, or strain**	16.1%	15.3%	15.9%	
**Upper extremity fracture**	17.7%	20.5%	17.6%	
**Upper extremity dislocation, sprain, or strain**	14.3%	15.1%	13.7%	
**Upper extremity open wound**	6.0%	6.1%	5.8%	
**Upper extremity superficial**	5.0%	4.8%	4.9%	
**Lower extremity fracture**	17.2%	18.0%	18.1%	
**Lower extremity dislocation, sprain, or strain**	24.6%	21.9%	25.2%	
**Lower extremity open wound**	4.0%	3.9%	3.9%	
**Lower extremity superficial**	6.6%	6.3%	6.5%	
**Other injury region/nature**	17.1%	19.3%	17.4%	
**Unadjusted EQ-5D social tariff**	0.94	0.75	0.78	0.82

Percentages may not add to 100 due to rounding.For injury types, percentages do not add to 100 as each individual can have more than one injury type recorded.

Recovery status was reported by 1248 participants at the 5-month interview, of whom 287 (23%) were fully recovered, and by 1937 participants at the 12-month interview, of whom 706 (36%) were fully recovered. We used two measures of recovery – *i.e.* participants’ reports of having “completely recovered” and having attained at least the same WHODAS level – as each measure on its own may miss some important aspects of recovery. For example, at the 12-month interview, 970 participants reported that they had “completely recovered” and 1025 reported they had attained at least the same level of functioning as pre-injury on all WHODAS dimensions, but only 706 passed both these tests.

The most common injury types were: spine dislocation, sprain, or strain; upper extremity fracture; upper extremity dislocation, sprain, or strain; lower extremity fracture; and lower extremity dislocation, sprain, or strain. Note that the injury data record more than one injury type for many participants, so the percentages do not add to 100.

The mean (unadjusted) EQ-5D health state value for the general population was 0.82. For the POIS cohort, their mean recalled pre-injury value was 0.94, falling to 0.75 and 0.78 5 and 12 months after injury respectively, where all three estimates are statistically significantly different than the general population mean (p < 0.001).

### Pre- and post-injury health status

If recalled pre-injury health valuation is unbiased, we would expect that: (1) pre-injury health state values are statistically the same as post-injury values for fully recovered participants, and (2) pre-injury health state values are significantly higher than post-injury values for non-recovered participants. We found a small but statistically significant positive difference for participants who had fully recovered, and a large positive difference for participants who had not fully recovered (Table 2). These differences were consistent when measuring recovery at both 5-months and 12-months post-injury.

**Table 2:** EQ-5D HRQoL pre-injury and at 5- and 12-months post-injury, by recovery status

**Recovery status at 5-months post-injury:**	**Pre-injury**	**5-months post-injury**	**Difference**
**Recovered (n = 287)**	0.98	0.95	0.03
	(0.97, 0.99)	(0.94, 0.96)	(0.01, 0.04)
**Not Recovered (n = 961)**	0.93	0.70	0.23
	(0.92, 0.94)	(0.69, 0.71)	(0.22, 0.25)
**Recovery status at 12-months post-injury:**	**Pre-injury**	**12-months post-injury**	**Difference**
**Recovered (n = 706)**	0.96	0.93	0.04
	(0.96, 0.97)	(0.92, 0.94)	(0.02, 0.05)
**Not Recovered (n = 1231)**	0.93	0.71	0.22
	(0.93, 0.94)	(0.70, 0.72)	(0.21, 0.24)

95% confidence intervals in parentheses.

Columns may not add to totals due to rounding.

### Health status of POIS participants and the general population

To test the validity of using population norms as a proxy for pre-injury health, we compared age- and sex-adjusted population norms with POIS participants’ health status before and after injury, by recovery status. Both the recovered and non-recovered groups had significantly better recalled pre-injury health than the corresponding New Zealand norm (Table 3 – upper panel). Participants who had fully recovered also reported significantly higher post-injury health than the general population, while the non-recovered reported significantly lower health values than the general population (Table 3 – lower panel).

**Table 3:** EQ-5D HRQoL by recovery at 5- and 12-months post-injury and age- and sex-adjusted NZ norm

	**Recalled pre-injury EQ-5D HRQoL**	**NZ norm**	**Difference**
**Recovered at 5-months (n = 287)**	0.98	0.85	0.12
	(0.97, 0.99)	(0.84, 0.87)	(0.11, 0.14)
**Not Recovered at 5-months (n = 961)**	0.93	0.85	0.09
	(0.92, 0.94)	(0.84, 0.86)	(0.07, 0.10)
**Recovered at 12-months (n = 706)**	0.96	0.86	0.10
	(0.96, 0.97)	(0.85, 0.87)	(0.09, 0.12)
**Not Recovered at 12-months (n = 1231)**	0.93	0.85	0.09
	(0.93, 0.94)	(0.83, 0.86)	(0.07, 0.10)
	**Current EQ-5D HRQoL**	**NZ norm**	**Difference**
**At 5-months post-injury:**			
**Recovered (n = 287)**	0.95	0.85	0.10
	(0.94, 0.96)	(0.84, 0.87)	(0.08, 0.11)
**Not Recovered (n = 961)**	0.70	0.85	−0.15
	(0.69, 0.71)	(0.84, 0.86)	(−0.16, −0.13)
**At 12-months post-injury:**			
**Recovered (n = 706)**	0.93	0.86	0.07
	(0.92, 0.94)	(0.85, 0.87)	(0.05, 0.08)
**Not Recovered (n = 1231)**	0.71	0.85	−0.14
	(0.70, 0.72)	(0.83, 0.86)	(−0.15, −0.12)

95% confidence intervals in parentheses.

Columns may not add to totals due to rounding.

## Discussion

Our results show that both retrospectively measured pre-injury health status and population norms differ from the health status reported by participants who had fully recovered from injury. The difference is greater between population norms and recovered health status than between recalled pre-injury status and recovered status. These findings are consistent with patients’ recall of their health status prior to injury exhibiting a small upward bias, and the general population being unrepresentative of those who are injured.

These results contrast with those of Watson and colleagues [8], who found that for completely recovered patients their retrospectively measured pre-injury health state values closely matched their values 12-months after injury using the SF-6D, although they found a marginally significant difference using the AQoL. A likely explanation for this difference is the increased statistical power available in our tests due to the larger sample size of the POIS cohort (1937 reporting recovery status at the 12-month interview compared to 186 for Watson *et al.*). However, there is also a possibility that recall bias may have been more pronounced in our study due to our 3-month delay between the injury event and measurement of recalled pre-injury health status.

Several studies have found that the general population may not be representative of populations of ill or injured individuals in terms of pre-onset health status [13-15]. Some populations, such as people with hip fracture [16], are in poorer health than the general population prior to their injury, whereas others, for example gunshot victims [17], are in better health. This article’s findings support the view that those who are injured are generally healthier than the general population.

An alternative explanation for the observed difference between self-reported health and the general population norm is ‘response shift’. This is the theory that individuals’ reference points for health status valuations change as their health changes [18]. Study participants, having had experience with a poorer health state due to injury, may tend to inflate their assessments of both their pre-injury and recovered health states by implicit comparison with their injured state. Without the opportunity to undertake prospective evaluations of pre-injury health, it is not possible to test for the presence of response shift. Schwartz and Sprangers [19] argue that the existence of response shift implies that the use of recalled health status is more appropriate than prospective measurement for evaluating the impact of health state changes on HRQoL, as recalled and current status are both completed with the same internal standard of measurement (*i.e.* experience with the new health state). This argument also implies that recalled pre-injury evaluation should be used instead of population norms to assess changes in health status, as the general population has not had the same injury experience as the study population.

Implicit theories of memory may help to explain the small bias found in recalled health status [20]. One important implicit theory in this context focuses on the stability of perceptions of self. Though people generally assume consistency in their personal attributes, significant events – such as injury – can provide a context in which recall is altered. Without a suitable reference point with which to recall their pre-injury health, people may begin by assessing their current health status and then adjusting that status for the expected change due to injury. If people tend to overestimate the change caused by injury, retrospective evaluation will be biased upward compared to actual pre-injury health. Our results provide some evidence in support of this theory, although the estimated effect is small.

## Conclusion

Retrospective evaluation of pre-onset health status is likely to be more appropriate than applying population norms to measure the effects of acute-onset illness or injury on HRQoL, although users of this approach should be aware of the potential for a small upward bias in such measurements.

## Abbreviations

POIS: Potential Outcomes of Injury Study; SF-6D: Short Form 6-dimension health status instrument; EQ-5D: EuroQol 5-dimension health status instrument; AQoL: Assessment of Quality of Life health status instrument; QALY: Quality-adjusted life year; NICE: National Institute for Health and Clinical Excellence; PROMS: Patient Reported Outcomes; HRQoL: Health-related quality of life; ACC: Accident Compensation Corporation; WHO: World Health Organization; WHODAS 2.0: World Health Organization Disability Assessment Schedule.

## Competing interests

All authors declare that they have no competing interests that may be relevant to the submitted work.

## Authors’ contributions

RW led the preparation of this article with support from SD, and conducted the statistical analysis. SD leads the POIS research team. PH and JL are POIS co-investigators. All authors contributed to the writing and editing of the manuscript. RW is guarantor. All authors read and approved the final manuscript.
